# Association between Hand Hygiene Knowledge and Self-Efficacy in Nursing Students: A Multicenter Cross-Sectional Study within the Framework of the Erasmus Project

**DOI:** 10.3390/nursrep14030147

**Published:** 2024-08-11

**Authors:** Ljudmila Linnik, Nuray Turan, Cansu Polat Dünya, Kati Lahtinen, Teija Franck, Maija Valta, Tuluha Ayoğlu, Nuray Akyüz, Verónica Coutinho, Luis Paiva, Irma Brito, Natura Colomer-Pérez, María del Carmen Giménez-Espert, Cristina Buigues, Omar Cauli

**Affiliations:** 1 Tallinn Health Care College, 13418 Tallinn, Estonia; ljudmila.linnik@ttk.ee; 2 Department of Fundamentals of Nursing, Faculty of Nursing, Istanbul University, 34116 Istanbul, Turkey; nkaraman@istanbul.edu.tr; 3 Department of Internal Medicine Nursing, Faculty of Nursing, Istanbul University, 34116 Istanbul, Turkey; cansu.polat@istanbul.edu.tr; 4 Faculty of Health and Well-Being, Turku University of Applied Science, 20520 Turku, Finland; kati.lahtinen@turkuamk.fi (K.L.); teija.franck@turkuamk.fi (T.F.); maija.valta@turkuamk.fi (M.V.); 5 Florence Nightingale Faculty of Nursing, Istanbul University-Cerrahpasa, 34381 Istanbul, Turkey; tuluha@iuc.edu.tr (T.A.); nuray.akyuz@iuc.edu.tr (N.A.); 6 Escola Superior de Enfermagem de Coimbra & UICISA:E, 3046-851 Coimbra, Portugal; vcoutinho@esenfc.pt (V.C.); luispaiva@esenfc.pt (L.P.); irmabrito@esenfc.pt (I.B.); 7 Department of Nursing, University of Valencia, 46010 Valencia, Spain; natura.colomer@uv.es (N.C.-P.); maria.c.gimenez@uv.es (M.d.C.G.-E.); cristina.buigues@uv.es (C.B.)

**Keywords:** infection control, nursing, nursing skills, education

## Abstract

Adherence to hand hygiene procedures is crucial for all populations, and the World Health Organization (WHO) has implemented specific guidelines for infection control. Frequent and correct hand hygiene can prevent infections, but non-compliance with hand hygiene is pervasive. Nursing students address this issue from the beginning of their training. In nursing training, self-efficacy is crucial in enhancing students’ competence, motivation, and clinical performance. We performed a cross-sectional multicenter study in five European countries, with a cross-sectional design with an online application of an instrument measuring hand hygiene knowledge based on WHO guidelines and general self-efficacy and specific self-efficacy for infection control. A total of 638 first-year nursing students participated in this study. The mean percentage of correct answers was 67.9%, with a considerable difference depending on the items. The worst results were obtained for questions related to sources of infection and types of hand hygiene methods in different situations. Finnish students displayed significantly (*p* < 0.001) higher scores in HH knowledge, whereas Estonian students had significantly (*p* < 0.001) higher levels of self-efficacy. There were significant correlations between the hand hygiene knowledge score and the self-efficacy score (*p* < 0.001). A multivariate analysis by linear regression analysis showed significant associations between the hand hygiene knowledge survey score and the students’ age (*p* < 0.001, OR = 0.18, 95% CI 0.04–0.10), as well as their country of origin (*p* = 0.01, OR = 0.09, 95% CI 0.03–0.34). HH knowledge is quite low among nursing students, and is correlated with self-efficacy, although the strongest predictors are age and country of origin. Different nursing curricula must favor HH knowledge, with varying degrees of emphasis depending on the country.

## 1. Introduction

Healthcare-associated infections continue to be a significant health challenge in Europe, with the daily incidence of healthcare-associated infections estimated at 6.5%, according to the most recent data from the European Centre for Disease Control and Prevention (ECDC) [1]. Every day, around 98,166 patients acquire an infection in a European healthcare facility, with 3.8 million patients affected annually, putting their lives at risk. According to another study by the World Health Organization (WHO), between 7% and 15% of hospitalized patients acquire at least one healthcare-associated infection, and 10% die^2^. However, at least 20% of these infections could be prevented through prevention and control programs and avoided with practices as simple as hand washing [2]. This evidence was presented by Florence Nightingale in her studies.

In this context, healthcare professionals have called for improved infection control and prevention in hospitals, and reinforced laboratory diagnostics, isolation capacity, hygiene protocols, and antimicrobial programs [3], in addition to the implementation of common protocols among member states and efforts to automate the detection and surveillance of these diseases. The implementation of complex strategies that include components such as system changes, education, awareness, promotion of patient safety culture, committed leadership, and positive reinforcement strategies, in addition to increased supervision and timely feedback, are considered crucial [3]. Likewise, European healthcare systems are seeking to highlight the importance of hand hygiene as a safe, simple, inexpensive, and effective practice to prevent and control infections in healthcare settings [4].

Another important aspect of hospital infection control and prevention in healthcare systems worldwide are nurses, who constitute the largest occupational group in the healthcare sector: there are 27.9 million nursing professionals, accounting for approximately 59% of healthcare professionals. They are also the professionals who spend the most time with patients and families, and therefore interact the most with them [5]. It is therefore important to stress the crucial role of their knowledge of hand hygiene and in the prevention and control of infection as a result. Another group that cannot be ignored are nursing students, who as future nurses also play a fundamental role in the prevention and control of infection [6,7,8,9,10], Hand washing plays a key role in reducing the transmission of infections [11], with the practice of hand hygiene being the most effective strategy in reducing the transmission of pathogenic microorganisms, costs, and the overall burden of healthcare-associated infections [12,13]. Nursing students should therefore receive regular guidance and supervision to attain the adequate knowledge, skills, and attitudes towards hand hygiene, and the literature shows studies that indicate shortcomings in the training of students in infection control and prevention in relation to hand hygiene [5,13,14,15,16]. This is in addition to non-compliance with hand hygiene standards in nursing students (76.4%) [17], a progressive decline in hand hygiene compliance as the academic year increases) [18], and difficulties or a lack of interest in applying theoretical knowledge in clinical practice [19]. There is also a need for repeated educational interventions and seminars on hand hygiene to promote positive attitudes and reinforce knowledge [20]. 

The principles of hand hygiene (HH) should be reiterated and emphasized periodically, not only to professionals but also to non-professionals [21]. In addition, the literature shows that studies analyzing HH practices among nursing students are limited, and less attention is paid to investigating their practices than those of nurses, without considering that they are the nurses of the future [20,22]. Moreover, the studies present different approaches in their formative contents, using samples of nursing students from different courses and with reduced samples. Multicenter studies are also scarce, with the consequent decline in the representativeness and generalization of the results [13,14,23].

In social cognitive theory, self-efficacy refers to a person’s belief in their capacity to successfully perform particular tasks or behaviors. In nursing education, self-efficacy plays a vital role in enhancing students’ competence, motivation, and clinical performance, which influences job satisfaction and the quality of the patient care provided [24,25]. Believing in one’s capacity to correctly perform these practices has a direct influence on adherence to these protective behaviors. When health professionals have high self-efficacy in relation to HH, they tend to engage in these practices more consistently and appropriately, reducing the risk of transmission of microbial contamination and contributing to a safer and healthier work environment [26].

This study was therefore conducted to ascertain the knowledge nursing students have about HH at the beginning of their studies and its relationship with self-efficacy. We thereby aimed to broaden our knowledge of this subject in order to study the phenomenon in various European countries (Estonia, Turkey, Portugal, Finland, and Spain) to be able to establish consensual and joint future training strategies. This will enable us to jointly address HH training and therefore infection control, in accordance with the guidelines established by international organizations [1,2].

## 2. Materials and Methods

### 2.1. Study Design

We conducted a cross-sectional study to determine the level of knowledge of HH and self-efficacy among nursing students from the Universities that participated in the setting of study (Erasmus + KA 220, entitled “Innovative, equally accessible teaching model for infection control: from nursing students to the general population” (grant number Erasmus plus KA220-HED-000100625) enrolled in the study during the last 4 months of the 2023. The project includes 6 Nursing faculties/schools from in European countries: the Faculty of Nursing at the University of Valencia (Valencia, Spain), Tervishoiu Kõrgkool (Tallinn, Estonia), the Faculty of Nursing (Istanbul University, Istanbul, Turkey), the Escola Superior de Enfermagem de Coimbra (Coimbra, Portugal), the Florence Nightingale Faculty of Nursing (Istanbul University-Cerrahpasa, Turkey) and Turun Ammattikorkeakoulu (Turku University of Applied Sciences, Turku, Finland). Eligible participants must be registered in the first year of a Bachelor of Nursing degree. The professors involved in the project invited all students by sending an e-mail to their institutional e-mail address to explain the project and inviting them to complete an anonymous questionnaire about their basic socio-demographic data (age, country, gender) and questionnaires to evaluate hand hygiene knowledge and self-efficacy.

### 2.2. Evaluation of Hand Hygiene Knowledge

All students who volunteered to answer the questionnaire were included in the study. A questionnaire developed by the WHO was used to assess the degree of perceptions/opinions and knowledge of HH validated in different languages. A general self-efficacy questionnaire [27] and a questionnaire to specifically assess self-efficacy in infection control [28] were used to assess self-efficacy.

The instrument used for the survey was the World Health Organization [29] hand hygiene questionnaire for healthcare workers. The items in the questionnaire were presented in English and translated into the local language. Question 1 covered training during the last three years, and question 2 covered compliance with the routine use of an alcohol-based solution. The last eight questions assessed the level of knowledge of hand hygiene.

We evaluated the percentage of right/wrong answers for each item (1–10). We also calculated a total score of HH knowledge ranging from 0 to 24, based on correct answers. A score of “1” was allocated to each correct answer, while “0” was given to each wrong answer. Items 1 and 2 were omitted from the calculation of the HH knowledge total score, since they do not refer to specific questions about hand hygiene knowledge.

### 2.3. Evaluation of Self-Efficacy

The general self-efficacy questionnaire is a 10-item scale that measures the self-reported self-efficacy with good internal reliability (Cronbach’s alpha of between 0.76 and 0.90) [27]. Each item is rated on a 4-point scale ranging from 1 (not at all true) to 4 (completely true). The total score was calculated by finding the total for all the items (ranging between 10 and 40, with a higher score indicating more self-efficacy). The items for assessing self-efficacy in infection control were six items related to infection control taken from the Health Belief Model (HBM) on Infection Control Perception [28]. ‘The Health Belief Model’ is a theory-based model used to predict health-related behaviors in assessing hospital clinical professionals’ perceptions and knowledge of infection control practices with an internal reliability for Cronbach’s alpha of between 0.65 and 0.81 [30]. Each item is rated on a 5-point scale, ranging from 1 (Strongly disagree) to 5 (Strongly agree). The higher the score, the greater the self-efficacy in infection control.

### 2.4. Ethical Issues

Permission to conduct this study was obtained from the university ethics committee of the University of Valencia (UV-INV_ETICA-2724568, approval on 1 June 2023), which leads this multi-center study. Information about the study was given to all the participants, and informed consent was obtained prior to completion of the questionnaires. Participation in the study was voluntary, and the participants were free to refuse to answer questions or withdraw from the study at any time. The participants were informed that their confidentiality would be protected by removing any identifying information from the interview transcripts, and that no individual would be identifiable in any reports detailing the study findings.

### 2.5. Sample Size Determination

The sample size was determined using a single population proportion formula based on the following assumptions: the level of HH knowledge among nursing students is 50%; (since no previous study has been conducted in the area related to the topic); a 95% confidence level; a 5% margin of error. The sample size calculated was 384.

n = (Zα/2)2p(1 − p)d2 = (1.96)20.5(1 − 0.5)(0.05)2 = 384

### 2.6. Data Collection

The data were collected electronically using a self-administered questionnaire specifically designed for this study, using Google Forms. The questionnaire contained three sections. The first section documented the students’ socio-demographics, such as age, gender, and marital status. The second section evaluated their HH knowledge based on the WHO questionnaire (tools for evaluations, WHO 2009). The third section evaluated their general self-efficacy (10 items) and self-efficacy related to infection control (6 items). The participants were first-year nursing students recruited between September 2023 and December 2023.

### 2.7. Statistical Analysis 

The results obtained for the quantitative variables are in the form of mean and standard deviation (mean ± SD). The quantitative variables were compared using a t-test or via the presence of abnormal distribution using the Mann–Whitney U test or Kruskal–Wallis test. The Bonferroni correction of the p value was adopted when significant differences were detected by the Kruskal–Wallis Test in order to counteract the family-wise error rate problem by adjusting the alpha value based on the number of tests. The categorical variables were compared by the chi-square test or Fisher’s exact test. Correlation among the quantitative variables was examined using the Pearson correlation coefficient and Spearman’s rank correlation coefficient. Multivariate logistic regression analysis was used to determine the differences in the indices in the presence of the basic characteristics of patients as confounding factors, and the results were stated as the odds ratio (95% confidence interval). SPSS Version 21 and SAS Version 9.1 were used for the statistical data analysis. The significance level was considered at least *p* < 0.05.

## 3. Results

### 3.1. Characteristics of the Study Sample

The research team sent the invitation to all first-year students of each faculty/school participating in the study. A total of 638 first-year nursing students participated in this study, of which 228 (35.7%) were from Spain, 167 (26.2%) from Turkey, 109 (17.1%) from Estonia, 68 (10.7%) from Portugal, and 66 (10.3%) from Finland. The average age of the participants was 21.7 ± 0.29 (SEM), and their ages ranged between 17 and 60 years. Regarding gender, the majority of participants, 531 in total (83.2%), identified as female, while 102 (16%) identified as male. Additionally, a small number identified as gender fluid (3; 0.4%), 1 as pangender (0.2%), and 1 as other (0.2%). Most of the participants, a total of 288 (45.1%), lived with their parents. Meanwhile, 163 (25.5%) lived in shared housing with other people, 45 (7.1%) lived with their partners, 37 (5.8%) lived with their partners and children, 25 (3.9%) lived with other family members or close friends, and 11 (1.7%) resided only with their children.

### 3.2. Knowledge of HH

The percentages of correct and uncorrected answers of HH knowledge based on the WHO questionnaire are shown in Table 1. When analyzing the differences in HH knowledge between nursing students from different countries, a significant effect emerged (*p* < 0.001), as the Finnish students’ knowledge was significantly better than that of the students in other countries. The mean level of correct answers in the study was 70%. However, this depends on the items in the HH knowledge survey. The worst results were reported for item 4 (“What is the most frequent source of germs responsible for healthcare-associated infections?”) with only 35.4% of the sample identifying the correct answer, item 7c (“Hand rubbing is more effective against germs than hand washing”), with 43.1% identifying the correct answer, and item 9 (“Which type of hand hygiene method is required in the following situations? Before giving an injection” (42.5% identified the correct answer), “After emptying a bedpan” (27.4% identified the correct answer), and “After removing examination gloves” (36.8% identified the correct answer)).

### 3.3. Self-Efficacy Score and Its Relationship with Knowledge of HH

The mean score for general self-efficacy was 30.54 ± 0.18 (SEM) (range 14–40), and the mean value of the self-efficacy score on infection control was 21.40 ± 0.19 (SEM) (range 6–30). When analyzing the differences in self-efficacy scores among nursing students from different countries, a significant effect emerged for both general self-efficacy and self-efficacy related to infection control (*p* < 0.05, with Bonferroni correction), with the results of Estonian students significantly better than for students in other countries. 

No significant correlations were observed between the hand hygiene knowledge survey score with the general self-sufficiency (GSE) questionnaire score (Rho = 0.02, *p* = 0.60, Spearman’s correlation). However, there were significant correlations between the hand hygiene knowledge survey and the infection control self-efficacy score (Rho = 0.21, *p* < 0.001, Spearman’s correlation). Furthermore, a significantly positive correlation was identified between the general self-efficacy questionnaire (GSE) score and the infection control self-efficacy score (Rho = 0.25, *p* < 0.001, Spearman’s correlation).

Significant differences were also observed between the general self-efficacy questionnaire score and gender (*p* < 0.01, Mann–Whitney test). However, no significant differences were found between the hand hygiene knowledge score and gender (*p* = 0.22, Mann–Whitney test). Furthermore, no significant differences were observed between infection control self-efficacy and gender (*p* = 0.24, Mann–Whitney test).

### 3.4. Multivariate Analyses

A linear regression analysis was conducted to examine the associations between hand hygiene knowledge score and various predictor variables, including age, gender, country, general self-efficacy, and infection control self-efficacy. The results indicated significant associations between hand hygiene knowledge score and age (*p* < 0.001, OR = 0.18, 95% CI 0.04–0.10), as well as the country of origin (*p* = 0.01, OR= 0.09, 95% CI 0.03–0.34). Furthermore, significant associations were observed with the infection control self-efficacy score (*p* < 0.001, OR = 0.20, 95% CI 0.06–0.18). However, no significant associations were found with other variables, including gender (*p* = 0.46, OR = −0.02, 95% CI −0.67–0.30), general self-efficacy scale score (*p* = 0.48, OR = 0.04, 95%CI −0.04–0.09), or the general self-efficacy scale score categorized with the highest quartile (>34 points, indicating better general self-sufficiency) (*p* = 0.16, OR = 0.07, 95% CI −0.20–1.24).

A second linear regression analysis was performed to examine the association between HH knowledge and each item on the infection control self-efficacy scale. The results indicated significant associations with 4 of the 6 items of infection control self-efficacy (Table 2). Specifically, significant associations were found in the items that referred to whether they sought information about infection control practices (*p* < 0.0018, OR = 0.13, 95% CI 0.09–061), whether they considered it important to adopt appropriate infection control measures (*p* = 0.003, OR = 0.18, 95% CI 0.15–0.76), if they regularly followed infection control recommendations (*p* < 0.0012, OR = −0.16, 95% CI −0.72–−0.16), and if they often used hand sanitizer while working in the healthcare setting (*p* = 0.04, OR = −0.10, 95% CI 0.001–0.51). However, no significant associations were found with the remaining items, as shown in Table 2.

## 4. Discussion

This research involved conducting a comparative analysis of nursing students’ knowledge of hand hygiene across five different countries, and correlating it with general infection control principles and specific self-efficacy. Such international comparisons provide valuable insights into how varying educational and practical conditions impact nursing students’ awareness of hand hygiene and self-efficacy [14,31]. The countries included in the study are associated with an ERASMUS collaborative project aimed at developing a common learning module on infection control principles for students in five different European countries, ensuring a high level of quality in healthcare delivery.

The results of this research are valuable on multiple levels. First, they provide essential input for curriculum development and enhancement in the field of hand hygiene. Understanding nursing students’ knowledge and self-efficacy in different countries means that teaching courses can be tailored or created according to specific needs. Second, this research uses the World Health Organization (WHO) hand hygiene questionnaire to assess nursing students’ knowledge levels, as this questionnaire widely used to assess HH knowledge among professional staff [6,32,33,34]. This ensures the study’s reliability and enables comparison with other research findings. The results indicate that nursing students’ hand hygiene knowledge in the five countries is moderately aligned with WHO guidelines, which is consistent with previous studies by Shinde and Mohite [9], Blomgren et al. [6], and Cambil-Martin et al. [8]. Notably, the Finnish students’ results stand out, suggesting cultural and educational differences may play a role in the greater knowledge of HH in Finnish nursing students observed in our study. A study performed in secondary schools during the COVID-19 pandemic in Finland, the Netherlands, and Ireland [35] revealed that many students indicated that they felt responsible for contributing to halting the spread of the virus, and this feeling was strongest in Finland, where students explained that even in the mitigation relaxation periods, they still often chose not to meet up with their friends to remain responsible and protect their families. In the education field, Finland has consistently ranked high in the Program for International Student Assessment (PISA) [36]), which compares national educational systems internationally.

However, a more detailed analysis of items of the HH questionnaire reveals that all nursing students understand essential hand hygiene principles for patient protection in all countries analyzed in the present study. Previous studies [6,10] also support the findings of this study. Furthermore, this study found that several students mistakenly believed that the main sources of healthcare-associated infections are the patient’s surrounding environment rather than the patient’s microflora and healthcare personnel’s hands. This conclusion is consistent with similar findings by Blomgren et al. [6] and Shinde and Mohite [9].

The lack of knowledge regarding proper hand hygiene methods during injections, after removing protective gloves and after emptying a urinary bag is concerning. For instance, only 27.4% of the students in this study correctly answered that using hand antiseptic is sufficient when emptying a urinary bag. Similar results were found in the study by Silago et al. [10]. However, in the study by Blomgren et al. [6], 84% of the students knew the correct answer. These discrepancies suggest variations in knowledge levels related to hand hygiene principles, which may stem from different educational and practical conditions. Building on similar findings, Cambie-Martin et al. [8] concluded that the principles taught to students primarily focus on self-protection rather than protection of the patients. Singh and co-workers [37] further discuss the need for students to have a more in-depth understanding of the importance of hand hygiene in various clinical situations. They emphasize the need for enhancing training programs to ensure enhanced awareness and skillful implementation of hand hygiene practices in practice. 

The research findings indicate that nursing students’ knowledge of hand hygiene is associated with their self-efficacy in infection control. This illustrates that the deeper nursing students’ understanding of hand hygiene principles, the stronger their belief in their ability to implement infection control measures in their practical work. Erasmus et al. [38] highlighted the existence of negative models in practice, in which experienced healthcare workers do not adhere to hand hygiene guidelines. This illustrates how students may adopt unfavorable hand hygiene practices if they perceive them to be examples to follow. In the context of Bandura’s theory [38], one could argue that students’ belief in their ability to successfully perform certain actions is pivotal in their practical implementation, especially in situations with negative hand hygiene execution models. Differences in self-efficacy among different countries have been reported in other studies [39,40,41,42,43]. In our study, the students form the two Nordic countries, Estonia and Finland, had higher self-efficacy scores compared to students of southern countries (Spain, Turkey, and Portugal). One possible explanation for these results could be the higher socioeconomic status of Finland and Estonia, which has previously been associated with greater likelihood of categorization into higher self-efficacy profiles across various countries [42]. The results of a multigroup latent profile analysis with predictors suggested that a higher socioeconomic status is associated with an increased likelihood of being categorized into higher self-efficacy profiles across the three culturally different countries of United States, China, and Finland, which is consistent with prior studies [42,44]. Differences in social class could be internalized in the minds of students in the form of personal efficacy. Therefore, it could be useful for teachers to guide students, especially those belonging to lower socio-economic class, to believe that it is motivation and effort, rather than social origin, that could increase their self-efficacy and determine their future employment. Other factors such as student cognitive activation [45], teaching approaches [46], and school discipline and safety [47] could also contribute to the differences in self-efficacy among countries, which needs to be further explored as these factors can influence nursing curricula and thus nurses’ professional skills when these students join the healthcare system.

This study presents several limitations that should take into account; therefore, caution should be administered while generalizing the results of this study. This study’s cross-sectional design is a limitation per se, since it makes it difficult to establish cause-and-effect associations because it only reflects a one-time assessment of the alleged cause and effect. The time of the cross-sectional snapshot may not reflect the group’s typical or overall behavior. However, we have tried to mitigate this limitation by evaluating nursing students at the beginning of the academic year in the first year of a Bachelor of Nursing degree so the background differences might be due to cultural, social, and secondary school differences rather that differences in the Nursing curricula among countries. Another limitation is the issues associated with online surveys, including Internet access problems, response bias, survey fatigue, and the fact that we were not able to analyze the characteristics of students who did not participate in the study, so we cannot rule out that the non-participating students may have different characteristics from students who did participate in the study. However, the motivation to participate in the study should not have been biased by the professors’ judgement about these topics, since students knew before deciding to participate that the questionnaires were anonymous, as specified in the research protocol and approval by the Ethics Committee.

## 5. Conclusions

The authors of this study emphasize that although Finnish nursing students had the highest level of hand hygiene knowledge, this did not reflect a higher self-efficacy in infection control. Similar levels of self-efficacy were observed among Spanish, Turkish, and Portuguese students, indicating a potential uncertainty in applying knowledge in real clinical situations. This underscores the need to understand that the mere acquisition of knowledge does not guarantee proper adherence to hand hygiene; increasing self-efficacy is also crucial [37]. The findings highlight the need for continuous monitoring, evaluation, and updating of infection control materials. Traditional pedagogical methods may prove inadequate in enhancing self-efficacy. It is therefore essential to ensure that curricula are dynamic and meet the needs of students and evolving clinical requirements [6,39]. This enables nursing students to acquire not only the necessary knowledge but also self-efficacy and positive attitudes, which are essential for the practical application of hand hygiene in future medical practice. Engaging nursing students in academic environments and clinical settings is a challenging issue for nursing educators worldwide. In recent years, many nurse researchers have investigated various educational strategies to explore and develop the best ways to increase nursing students’ academic engagement. Different strategies could be established in nursing curricula across different countries in order to enhance HH knowledge among nursing students such as technology-based strategies, collaborative strategies, simulation based strategies, research based strategies, and miscellanea learning strategies [48,49]. But the role of modern technologies such as online Internet technologies and simulations are more attractive to students and make their participation more active in the learning process [49,50].

The data gathered in this study will be very useful in guiding the educational program and raising institutional awareness about strategies to increase early the level of HH knowledge to improve nursing students’ skills. The innovative, equally accessible teaching model for infection control will be applied to nursing students and to the general population. The instruments used in this initial assessment will be used after the training course, and as such, “before and after” data will increase evidence about effectiveness.

## Public Involvement Statement

No public involvement in any aspect of this research.

## Guidelines and Standards Statement

This study was performed in accordance with the Strengthening the Reporting of Observational Studies in Epidemiology (STROBE) Checklist (Cross-sectional studies).

## Use of Artificial Intelligence

AI or AI-assisted tools were not used in drafting any aspect of this manuscript.

## Figures and Tables

**Table 1:** Hand hygiene knowledge survey questionnaire for healthcare workers.

Questions	Frequency (%) of Possible Answers
Did you receive formal training in hand hygiene in the last three years?	
Yes	333 (52.2%)
No	305 (47.8%)
2. Do you routinely use an alcohol-based hand rub for hand hygiene?	
Yes	323 (50.6%)
No	315 (49.4%)
3. Which of the following is the main route of cross-transmission of potentially harmful germs between patients?	
(a) **Healthcare workers’ hands when not clean**.	322 (50.5%)
(b) Air circulating in the hospital.	79 (12.4%)
(c) Patients’ exposure to colonized surfaces (e.g., beds, chairs, tables, floors).	147 (23.0%)
(d) Sharing non-invasive objects (e.g., stethoscopes, pressure cuffs, etc.) between patients	90 (14.4%)
4. What is the most frequent source of germs responsible for healthcare-associated infections?	
(a) The hospital’s water system	5 (0.8%)
(b) The hospital air	103 (16.1%)
(c) **Germs already present on or within the patient**	226 (35.4%)
(d) The hospital environment (surfaces)	304 (47.6%)
5. Which of the following hand hygiene actions prevents transmission of germs to the patient?	
(a) Before touching a patient	
**Correct answer is “Yes”**	609 (95.5%)
(b) Immediately after a risk of fluid exposure	
**Correct answer is “No”**	507 (79.5%)
(c) After exposure to the immediate surroundings of a patient	
**Correct answer is “No”**	484 (75.9%)
(d) Immediately before a clean/aseptic procedure	
**Correct answer is “Yes”**	537 (84.2%)
6. Which of the following hand hygiene actions prevents the transmission of germs to the healthcare worker?	
(a) After touching a patient	
**Correct answer is “Yes”**	547 (85.7%)
(b) Immediately after a risk of body fluid exposure	
**Correct answer is “Yes”**	550 (86.2%)
(c) Immediately before a clean/aseptic procedure	
**Correct answer is “No”**	461 (72.3%)
(d) After exposure to the immediate surroundings of a patient	
**Correct answer is “Yes”**	530 (83.1%)
7. Which of the following statements on alcohol-based hand rub and hand washing with soap and water are true?	
(a) Handrubbing is more rapid for hand cleansing than hand washing	
**Correct answer is “True”**	444 (69.6%)
(b) Handrubbing causes skin dryness more than hand washing	
**Correct answer is “False”**	456 (71.5%)
(c) Handrubbing is more effective against germs than hand washing	
**Correct answer is “True”**	275 (43.1%)
(d) Handwashing and handrubbing are recommended to be performed in sequence	
**Correct answer is “False”**	504 (79%)
8. What is the minimal time needed for alcohol-based handrub to kill most germs on your hands?	
**20 s**	377 (59.1%)
3 s	16 (2.5%)
1 min	183 (28.7%)
10 s	62 (9.7%)
9. Which type of hand hygiene method is required in the following situations?	
(a) Before palpation of the abdomen	
**Rubbing**	376 (58.9%)
Washing	233 (36.5%)
None	29 (4.6%)
(b) Before giving an injection	
**Rubbing**	271 (42.5%)
Washing	358 (56.1%)
None	9 (1.5%)
(c) After emptying a bedpan	
**Rubbing**	175 (27.4%)
Washing	448 (70.2%)
None	15 (2.4%)
(d) After removing examination gloves	
**Rubbing**	235 (36.8%)
Washing	368 (57.7%)
None	35 (5.5%)
(e) After making a patient’s bed	
**Rubbing**	340 (53.3%)
Washing	272 (42.6%)
None	26 (4.1%)
(f) After visible exposure to blood	
Rubbing	219 (34.3%)
**Washing**	401 (62.9%)
None	18 (2.8%)
10. Which of the following should be avoided, as associated with increased likelihood of colonization of hands with harmful germs?	
(a) Wearing jewellery	
**Correct answer is “Yes”**	577 (90.4%)
(b) Damaged skin	
**Correct answer is “Yes”**	596 (93.4%)
(c) Artificial fingernails	
**Correct answer is “Yes”**	607 (95.1%)
(d) Regular use of hand cream	
**Correct answer is “No”**	632 (99%)

Nursing students from Portugal obtained the lowest mean scores for HH knowledge, followed by Spain and Turkey. Students from Finland displayed the highest level of HH knowledge (*p* < 0.05, with Bonferroni correction). The right answers to each question are marked in bold. Questions 1 and 2 do not have right or wrong answers.

**Table 2:** Association of HH knowledge with self-efficacy on infection control items. Significant *p* values are in bold.

Variable	Standardized Beta Coefficients	t	*p* Value	95% Confidence Interval Lower Limit–Upper Limit
I engage in good infection control practices	−0.003	−0.06	0.95	−0.33–0.31
I seek information on infection control practices	0.13	2.67	0.008	0.09–0.61
Engaging in proper infection control measures is important to me	0.18	2.93	0.003	0.15–0.76
I follow infection control recommendations regularly	−0.16	−3.09	0.002	−0.72–−0.16
I often use hand sanitizer while working in the healthcare setting	0.10	1.97	0.04	0.001–0.51
Hand sanitizers are as effective as hand washing in controlling infections	−0.007	−0.16	0.86	−0.20–0.17

## Data Availability

The data presented in this study are available on request from the corresponding author.

## Public Involvement Statement

No public involvement in any aspect of this research.

## Guidelines and Standards Statement

This study was performed in accordance with the Strengthening the Reporting of Observational Studies in Epidemiology (STROBE) Checklist (Cross-sectional studies).

## Data Availability Statement

AI or AI-assisted tools were not used in drafting any aspect of this manuscript.
